# *Pediococcus pentosaceus* JS35 improved flavor, metabolic profile of fermentation supernatant of mulberry leaf powder and increased its antioxidant capacity

**DOI:** 10.3389/fnut.2025.1551689

**Published:** 2025-03-04

**Authors:** Caiyan Meng, Yutong Wang, Jiawen Xie, Jiajia Xuan, Jingze Geng, Guanhui Liu, Jie Tu, Hang Xiao

**Affiliations:** ^1^College of Biotechnology, Jiangsu University of Science and Technology, Zhenjiang, Jiangsu, China; ^2^School of Grain Science and Technology, Jiangsu University of Science and Technology, Zhenjiang, Jiangsu, China; ^3^Department of Food Science, University of Massachusetts, Amherst, MA, United States

**Keywords:** *Pediococcus pentosaceus*, mulberry leaf, flavor, metabolic profiles, antioxidant capacity

## Abstract

*Pediococcus pentosaceus* JS35 was used to improve flavor, metabolic profile and antioxidant activity of mulberry leaf powder. Gas chromatography ion mobility spectrometry (GC-IMS) analysis revealed that fermentation increased the contents of floral and fruity flavor compounds such as dihydrolinalool and 2-phenylethanol, while decreased the grassy, pungent odor compounds. Non-targeted metabolomics analysis showed that *Pediococcus pentosaceus* JS35 altered the metabolic profile of mulberry leaf, especially increased the content of flavonoids metabolites such as kaempferol, quercetin and daidzein. Compared with the unfermented sample, the fermented supernatant had higher antioxidant capacity *in vitro* and in *Caenorhabditis elegans*. Furthermore, the fermented supernatant supplementation significantly prolonged the lifespan of *Caenorhabditis elegans*. In conclusion, fermentation by *Pediococcus pentosaceus* JS35 improved the flavor and active compounds of mulberry leaf, and the fermented product had effective antioxidant capacity. This study will provide ideas for the application of *Pediococcus pentosaceus* JS35 and the processing of mulberry leaf into functional foods or food ingredient.

## Graphical abstract

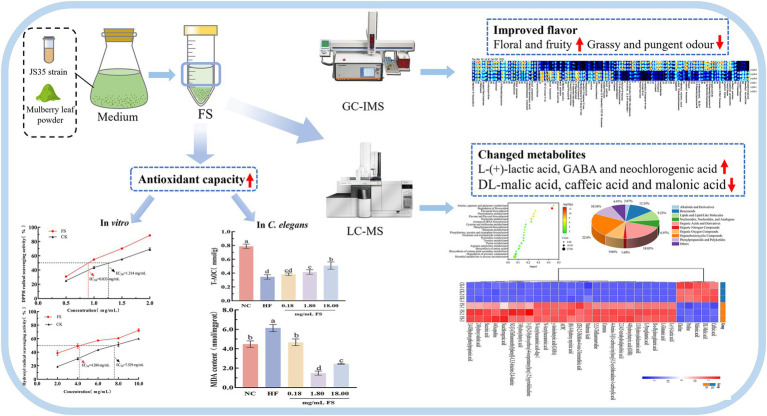

## 1 Introduction

Mulberry leaves are medicinal and edible containing functional components, such as polyphenols, polysaccharides, *γ*-aminobutyric acid (GABA) and alkaloids, etc., with potential antioxidant, fat-reducing, antibacterial and hypoglycaemic benefits (1). Recently, mulberry leaves have been increasingly used as functional food ingredient (2). The most well-known food prepared from mulberry leaves is mulberry leaf tea. Liu et al. (3) used mulberry leaf and fu brick tea as raw materials to prepare tea beverages, which reduced sugar absorption, oxidative stress and inflammatory response in diabetic rats, resulting in hypoglycemic effect. Mulberry leaves are also used to make yogurt, beverages and noodles, etc. (2). However, mulberry leaves contain high content of crude fiber, anti-nutritional factors and grassy flavor, which limits its further development and utilization (4). At present, the methods for improving the quality of mulberry leaves mainly include physical treatment, chemical treatment and microbial fermentation (4, 5). Among them, fermentation techniques have obvious advantages in terms of no solvent residues, loss of nutrients, and cost (6, 7).

Fermentation is an effective method to improve active ingredients, flavor and functional value of food materials. *Lactic acid bacteria* (LAB) are commonly used as safe fermentation start cultures. Hu et al. (8) used *Lactobacillus fermentum* and *saccharomyces cerevisiae* to ferment mulberry leaves to increase the dissolution and extraction of 1-deoxynojirimycin (DNJ). Tang et al. (9) used *Lactobacillus plantarum* to ferment mulberry pomace, and the 0.05 mg/mL extract from fermented mulberry pomace inhibited *α*-glucosidase by 93.41% *in vitro*, and also reduced glucose content and reactive oxygen species (ROS) level in *Caenorhabditis elegans* (*C. elegans*). In addition, it was reported that LAB fermentation reduced alcohols and aldehydes with grassy, beany and earthy flavors in mung beans and increased aromatic compounds with floral and fruity flavors, thereby improving the flavor of mung beans (10). Recently, there are some studies on microbial fermentation to improve the quality of mulberry leaves. Su et al. used *Pediococcus cellicola* and *Bacillus licheniformis* to ferment mulberry leaves and further investigated the effects of the fermented mulberry leaves (FML) on intestinal morphology, antioxidant capacity and immune function in finishing pigs. The results showed that FML significantly increased the nutrient digestibility of pigs, and supplementation with 10% FML increased the total antioxidant capacity of the ileum by 1-fold, as well as promoting antioxidant activity, immunity and intestinal mRNA expression levels in finishing pigs (11). Wang et al. (12) studied the liquid fermentation of mulberry leaves by *Monascus*. The results showed that the fermentation effectively improved the flavor of mulberry leaves and significantly up-regulated the volatile compounds with aromatic flavors such as (2R,3R) – (−)-2,3-butanediol, terpineol, and eugenol (12). Chen et al. (13) used a mixture microbial of *Lactobacillus plantarum*, *Candida utilis*, *Trichoderma koningii*, and *Bacillus cereus* for solid-state fermentation of mulberry leaves. After fermentation, the contents of total phenols and total flavonoids in mulberry leaves increased by 30 and 86%, respectively, and the antioxidant activity of mulberry leaves was significantly improved (13). During the fermentation process, the flavor of raw materials and the content of various active substances will be affected by various factors such as fermentation strains, strain ratio, fermentation method, fermentation time and temperature, etc. (14). Therefore, screening and optimizing specific microorganisms and controlling the fermentation conditions remain key issues in improving the quality of fermented mulberry leaves. Currently, most of the research on fermented mulberry leaves is used in the production of animal feed. The application in food production is mainly focused on the development of mulberry leaf tea, with less research in other forms of food development, which needs further research and vigorous promotion (5, 14). Moreover, there are still few specific LAB strains that can simultaneously improve the edible performance and active compound content of mulberry leaves. Screening and application of LAB strains from different sources are still important research topics for the high value utilization of mulberry leaves.

Mulberry leaves are a traditional feed for silkworms, and the intestinal flora of the silkworm plays an essential role in facilitating the absorption of nutrients from feed (15). In our previous study, a *Pediococcus pentosaceus* strain named JS35 was screened from silkworm intestine, and the strain was used to ferment mulberry leaves to increase GABA content (16). Further exploring the potential application of the strain and promoting the diversified development of mulberry leaves will be an interesting and valuable study.

*Pediococcus pentosaceus* (*P. pentosaceus*) strains are normally salt tolerant, non-motile, Gram-positive cocci with facultative anaerobic and carbohydrate degradation features (17). It has been reported that *P. pentosaceus* has antioxidant, cholesterol lowering, and immune functions (18). Plessas et al. (19) reported that a sour-dough bread prepared with *P. pentosaceus* SP2 had better in terms of acidity, organic acid content and resistance to spoilage, compared to natural fermentation sourdough bread prepared under the same conditions.

In this study, the fermentation supernatant of mulberry leaf powder was prepared by *P. pentosaceus* JS35, and the metabolite composition and flavor compounds were investigated by liquid chromatography-mass spectrometry (LC–MS) and gas chromatography ion mobility spectrometry (GC-IMS), respectively. Furthermore, the antioxidant capacities of the fermented supernatant (FS) were studied *in vitro* and in *C. elegans*. The results will provide experimental basis for the application of mulberry leaves and *P. pentosaceus* JS35 strain in processing of functional food or food ingredients.

## 2 Materials and methods

### 2.1 Materials and chemicals

*Pediococcus pentosaceus* JS35 is a self-screening strain and stored in China General Microbiological Culture Collection Center (Beijing, China). Mulberry leaf powder (120 mesh) was purchased from Bozhou Hongyang Pharmaceutical Sales Co., Ltd. (Bozhou, China). *C. elegans* strains N_2_ (wild-type) and *Escherichia coli* (*E. coli*) OP50 were gifted by Professor Dong Ying, Jiangsu University. *γ*-aminobutyric acid (GABA) standard (≥99%) was purchased from Sigma Aldrich (St Louis, MO, United States). 1,1-Diphenyl-2-picrylhydrazyl free radical (DPPH) was purchased from TCI Chemical Industry Development Co., Ltd. (Shanghai, China). Ltd. (Shanghai, China). The total antioxidant capacity (T-AOC) assay kit and malondialdehyde (MDA) assay kit were purchased from Nanjing Jiancheng Bioengineering Institute (Nanjing, China). Other chemical reagents were analytical grade and purchased from Sinopharm Chemical Reagent Co., Ltd. (Shanghai, China).

### 2.2 Preparation of supernatant of mulberry leaves powder

The fermented supernatant (FS) was prepared by *P. pentosaceus* JS35. The strain was reactivated twice by culturing it at 37°C for 16 h each time in De Man, Rogosa, and Sharpe (MRS) broth before use. The experimental design consisted of two groups: (1) Control supernatant group (CK), each 100 mL of the solution contains 1.5 g of mulberry leaf powder. (2) Fermented supernatant group (FS). Each 100 mL of the solution contains 1.5 g of mulberry leaf powder, 1.5 g of L-glutamic sodium (L-MSG), and 1 g of glucose. Both groups were sterilized at 121°C for 20 min. The FS group was inoculated with 2 mL *P. pentosaceus* JS35 (10^7^–10^8^ CFU/mL, v/v), while the CK group was inoculated with distilled water of the same volume. Both groups were incubated at 30°C for 48 h. Subsequently, the medium was fully shaken and centrifuged at 8000 rpm for 10 min. The supernatant was collected for further determination.

### 2.3 Determination of physicochemical properties of supernatant

The pH of the samples was measured directly by a calibrated pH meter produced by Mettler toledo (Shanghai) Co., Ltd. (Shanghai, China). Total titratable acidity (TTA) content was measured with reference to GB12456-2021 “Determination of total acid in food.”

Determination of GABA content followed the method of Tang et al. (20). In brief, 1.0 mL of sample was added to 1.0 mL of 0.1 mol/L sodium tetraborate buffer, 1.2 mL of 6% (w/v) redistilled phenol solution, and 0.6 mL of 7% (v/v) sodium hypochlorite solution, mixed well, heated in a boiling water bath for 10 min and immediately took an ice bath for 5 min. When it appeared blue-green, 2.0 mL of 60% (v/v) ethanol solution was added. The absorbance at 640 nm was determined. The standard curve was prepared using 0 ~ 0.5 mg/mL GABA (*y* = 1.8852*x* −0.0185, *R*^2^ = 0.9987).

Total phenolics content (TPC) was determined according the method of Jin et al. (21). In brief, 1.0 mL sample was mixed with 1.0 mL Folin–Ciocalteu reagent and 2.0 mL of 10% (w/v) Na_2_CO_3_ solution, then diluted to 10.0 mL with distilled water and put in a water bath at 40°C for 1 h. Absorbance at 760 nm was measured. Different concentrations of pyrogallic acid were used for preparing a standard curve (0 ~ 0.10 mg/mL, *y* = 9.5573*x* + 0.1281, *R*^2^ = 0.9985).

Total flavonoids content (TFC) was determined according the method of Zhuo et al. (16). 0.3 mL of 5% NaNO_2_ was added to 2 mL of sample, then shacked well and stood for 6 min, then 0.3 mL of 10% AlCI_3_ solution was added, then shacked well and stood for 6 min. Finally, 2 mL of 4% NaOH solution was added, shacked well and then diluted to 10 mL with 40% ethanol solution. The mixture stood for 10 min and then the absorbance at 510 nm was measured. Different concentrations of rutin were used for preparing a standard curve (0 ~ 0.5 mg/mL, *y* = 1.2953*x* + 0.0137, *R*^2^ = 0.9993).

### 2.4 Sensory evaluation

The sensory characteristics of the samples from the CK and FS group were evaluated by quantitative descriptive analysis (QDA) according to the method of Guan et al. (22) with minor modifications. The group sensory test was performed using a 10-point scale (with 1 indicating “very bad” and 10 indicating “excellent”), including the following indicators: color, flavor, sourness, fermented, grassy odor, and overall acceptance (the details are shown in Supplementary Table S1). The tasting group consisted of 10 graduate students of food processing and safety from Jiangsu University of Science and Technology, who had the professional sensory ability to identify and describe food flavors. Appropriate protocols for protecting the right and privacy of all participate were utilized during the study, including the safety of raw materials and test samples was strictly controlled by the organizers, the study requirements and risks were full disclosure, no release of participants’ knowledge, and the right to leave from the sensory evaluation at any time. The sensory analysis was approved by the College of Biotechnology, Jiangsu University of Science and Technology. All participants signed informed forms.

### 2.5 Determination of volatile organic compounds by GC-IMS

The HS-GC–IMS analysis was conducted on the FlavourSpec^®^ GC-IMS device (G.A.S. Dortmund, Germany). Two gram of each sample was put into a 20 mL headspace bottle and incubated at 60°C for 20 min. Subsequently, 500 μL of the headspace sample was automatically injected into the syringe (no shunt mode) through a heated syringe at 85°C. The volatile organic compounds were separated by an MXT-5 capillary column (15 m × 0.53 mm). The column temperature was maintained at 60°C, and the IMS temperature was 45°C. Gas chromatography conditions: nitrogen as carrier gas, 0–10 min flow rate of 2 mL/min, 10–20 min flow rate of 10 mL/min, 20–30 min flow rate of 100 mL/min, the final flow rate increased to 150 mL/min.

The retention index (RI) of volatile compounds was calculated with C_4–_C_9_ as the external reference. The NIST database and IMS database built in the application software can be used for qualitative and quantitative analysis according to peak intensity.

### 2.6 Non-targeted metabolomics analysis

An UltiMate™ 3000 rapid separation (RS) HPLC system (Thermo Scientific, Waltham, MA, United States) with an electrospray ionization source was used for LC–MS/MS analysis. An ACQUITY UPLC HSS T3 C_18_ column (100 × 2.1 mm, 1.8 μm; Waters) was used for chromatographic analysis. The system conditions were as follows: column temperature 50°C, injection volume 1 μL, flow rate 0.4 mL/min. In the positive ion mode, the mobile phase consisted of 0.1% formic acid aqueous solution (v/v, A) and 0.1% formic acid acetonitrile solution (v/v, B). In negative ion mode the mobile phase consisted of water (A) and acetonitrile (B). Gradient elution: 0–0.5 min, 95% A; 0.5–5.5, 95–50% A; 5.5–9, 50–5% A; 9–10.5, 5% A; 10.5–10.6, 5–95% A; 10.6–12, 95% A. The mass spectrometry analysis was conducted under the following conditions: the sample signal was scanned at 67–1000 m/z, and the heating temperature of the ion source was set at 325°C. In both positive and negative ion modes, the ion spray voltage was set to 3.5 and 2.5 kV, respectively. The resolution was set to 30,000. To check the stability, the QC samples were continuously inserted.

Metabolites were identified by mass spectrometry matching. The original data were subjected to baseline filtering, peak identification, integration, retention time correction and peak alignment using the metabolomics processing software Compound Discoverer (Thermo Scientific, Waltham, MA, United States).

### 2.7 Antioxidant capacity *in vitro* assay

The DPPH radical scavenging capacity was assayed using the method described by Jin et al. (21). The lyophilized samples were reconstituted with distilled water to 0.5 ~ 2.5 mg/mL.

Hydroxyl radical scavenging capacity assay was determined according to the method of Joseph et al. (23) with a slight modification. Equal amounts of 9 mM salicylic acid–ethanol, 9 mM ferrous sulfate, sample solution and 0.1% (v/v) hydrogen peroxide were added in sequence and incubated at 37°C for 30 min. The mixed solution was centrifuged at 7,000 rpm for 3 min, and then the absorbance value was determined at 510 nm. Distilled water was used instead of the sample solution in the control group (*A*_max_), distilled water was used instead of hydrogen peroxide solution as the blank group (*A*_0_), and Vitamin C was used in a positive control.

Total reducing power was assayed using the method described by De Marino et al. (24) with a slight modification. 1.0 mL of sample solution was added to 1.0 mL of phosphate buffered saline (PBS, 200 mM, pH 6.6), and then 1.0 mL of 1% (w/v) potassium ferricyanide was added. The mixture was incubated at 50°C for 20 min and then 1.0 mL 10% (w/v) trichloroacetic acid was added. The mixture was centrifuged at 3000 rpm for 10 min. Then 1.0 mL of the supernatant was successively mixed with 1.0 mL of distilled water and 0.2 mL of 0.1% (w/v) FeCl_3_ and then incubated for 10 min at room temperature. The absorbance was measured at 700 nm. Vitamin C was used as a positive control.

### 2.8 Effects of FS on *C. elegans*

#### 2.8.1 *C. elegans* culture and experimental grouping

*Caenorhabditis elegans* were cultured on nematode growth medium (NGM) at 20°C and fed *E. coli* OP50 as food. When there were plenty of unhatched eggs on NGM and also inside of the nematode bodies, the nematodes reached gravid status. Then, the gravid nematodes were lysed in alkaline lysis solution (0.4 mL of M9 buffer, 0.4 mL of 1 M NaOH and 0.2 mL of 7% NaClO) to obtain age-synchronized eggs for subsequent experiments (25).

The experimental *C. elegans* were divided into normal control group (normal medium, NC), high-fat diet group (high cholesterol medium, HF), and different concentrations of FS treatment group (0.18, 1.80, 18.00 mg/mL FS, high cholesterol medium). Nematodes synchronized to L4 stage were randomly assigned to each group of plates and grown in an incubator at 20°C. The experiment group used FS mixed with *E. coli* OP50 at a ratio of 1:1 as the food source, while the control group was given distilled water mixed with *E. coli* OP50.

#### 2.8.2 Determination of T-AOC and MDA content in *C. elegans*

The *C. elegans* of above groups were transferred to NGM plates containing 12.5 mg/mL 5-fluorodeoxyuridine (FUDR) to prevent reproduction, and then the nematodes were transferred to fresh NGM plates (FUDR) every 2 days. Nematodes were collected after 4 days of treatment and washed with M9 buffer 3 times. Nematode samples were homogenized in an ice bath using a grinding pestle and then centrifuged at 7,000 rpm for 3 min at 4°C, and the supernatants were stored for assays.

The levels of T-AOC and MDA in *C. elegans* samples were measured according to the instructions of the T-AOC and MDA detection kits, respectively.

#### 2.8.3 Determination of growth indicators of *C. elegans*

Determination of lifespan according to the method of Zhou et al. (26), with a slight modification. Synchronized L4 larval stage *C. elegans* were transferred to NGM plates containing 12.5 mg/mL FUDR. Each group consisted of 30 nematodes and was cultured at 20°C. The survival and mortality of nematodes were recorded every 2 days. The living nematodes were transferred to fresh NGM plates until all nematodes were deceased. The death of nematodes was determined by their lack of response to repeated touching with a platinum wire.

The determination method of reproductive ability is as follows: the *C. elegans* of L4 stage were transferred to fresh NGM every day, until the end of oviposition. The oviposition plate was placed in an incubator at 20°C for 24 h, and the number of offspring was counted as the oviposition amount of nematodes. Each group of 10 nematodes was performed in parallel, and the experiment was repeated three times.

The determination method of head swing is as follows: the *C. elegans* synchronized to L4 stage were cultured in the corresponding NGM plates with 12.5 mg/mL FUDR. The head swing of nematodes was measured on the 5th and 10th day of treatment. A single nematode was selected and placed in the M9 buffer, which was balanced for 1 min. The number of head swings of nematodes within 30 s was observed using a stereoscope, and 30 nematodes were counted in each group (a head swing is when the head of the nematode swings from one side to the other, and then swings back again).

### 2.9 Statistical analysis

All sample reactions were performed in triplicate, and the results were expressed using the mean values ± standard deviation (SD). Duncan’s multiple comparison tests were performed to identify the difference between values using IBM SPSS Statistics 27.0 (SPSS Inc., Chicago, IL, United States). *p* < 0.05 was considered to indicate a statistically significant difference.

## 3 Results and discussion

### 3.1 Chemical properties and compounds of supernatant

**Figure 1:**
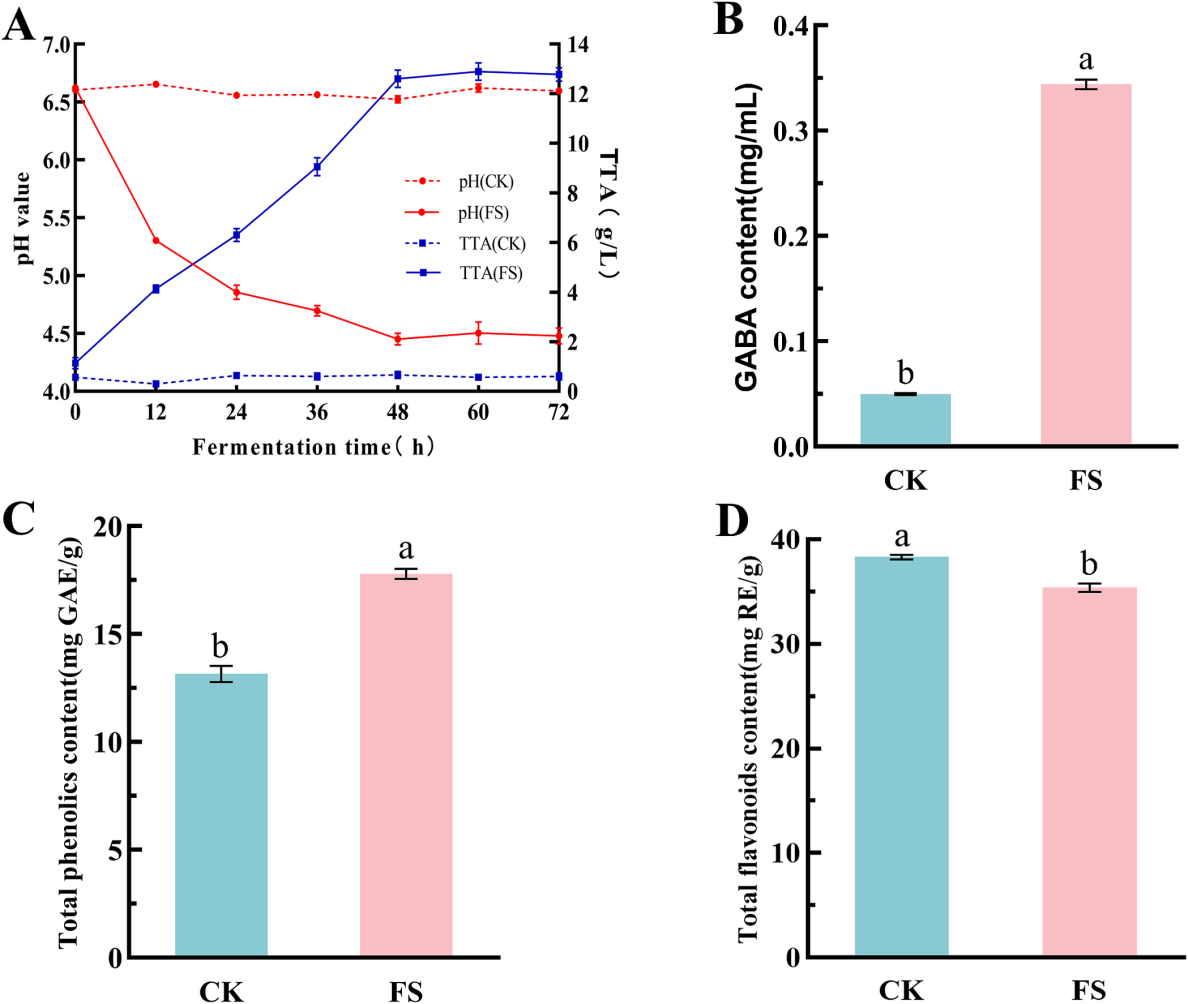
Effect of lactic acid bacteria fermentation on chemical composition of mulberry leaf powder. **(A)** Changes of pH and TTA during fermentation. **(B)** GABA content. **(C)** Total phenolics content. **(D)** Total flavonoids content. The letters a, b indicated significant differences in content of GABA, total phenolics and total favonoids contents (*p* < 0.05).

The changes in pH value, TTA, GABA, total phenolics and total flavonoids in different supernatants were determined. As shown in Figure 1. After 48 h of fermentation, the pH value decreased significantly, and the TTA increased significantly (*p* < 0.05) (Figure 1A). This was due to the accumulation of organic acids such as lactic acid and acetic acid produced by *P. pentosaceus* JS35 during fermentation (27).

GABA is an active compound in mulberry leaves (28), and it is also a food functional factor that has attracted much attention in recent years. *P. pentosaceus* JS35 is a GABA-producing strain isolated from the intestine of silkworms in our previous study (16). Compared to the unfermented supernatant, the GABA content in the FS group increased by 5.8-fold after 48 h of fermentation (Figure 1B). Some LABs have been reported having the potential to convert glutamic acid to GABA (29). The results showed that *P. pentosaceus* JS35 played the role well to produce GABA in the fermentation system containing mulberry leaf powder and L-MSG.

Phenolics and flavonoids are mainly focused bioactive components in mulberry leaves, which have many physiological functions such as antioxidation and hypoglycemia (30). In Figures 1C,D, the total phenolics and total flavonoids contents in the FS were 17.79 ± 0.23 mg GAE/g and 35.38 ± 0.41 mg RE/g, respectively. Compared to the unfermented supernatant, the total phenolics content increased by 35.29%, while the total flavonoids content decreased by 8.28%. LAB can produce some esterase to hydrolyze some bound phenolics during fermentation, and some complex macromolecular phenolics can be converted into small molecular phenolics, resulting in an increase in activities (31). There are few reports about the decreasing of flavonoids by LAB fermentation, however, it has been reported that the reduction of flavonoids during fermentation did not lead to a decline in biological activity (32).

### 3.2 Sensory evaluation analysis

**Figure 2:**
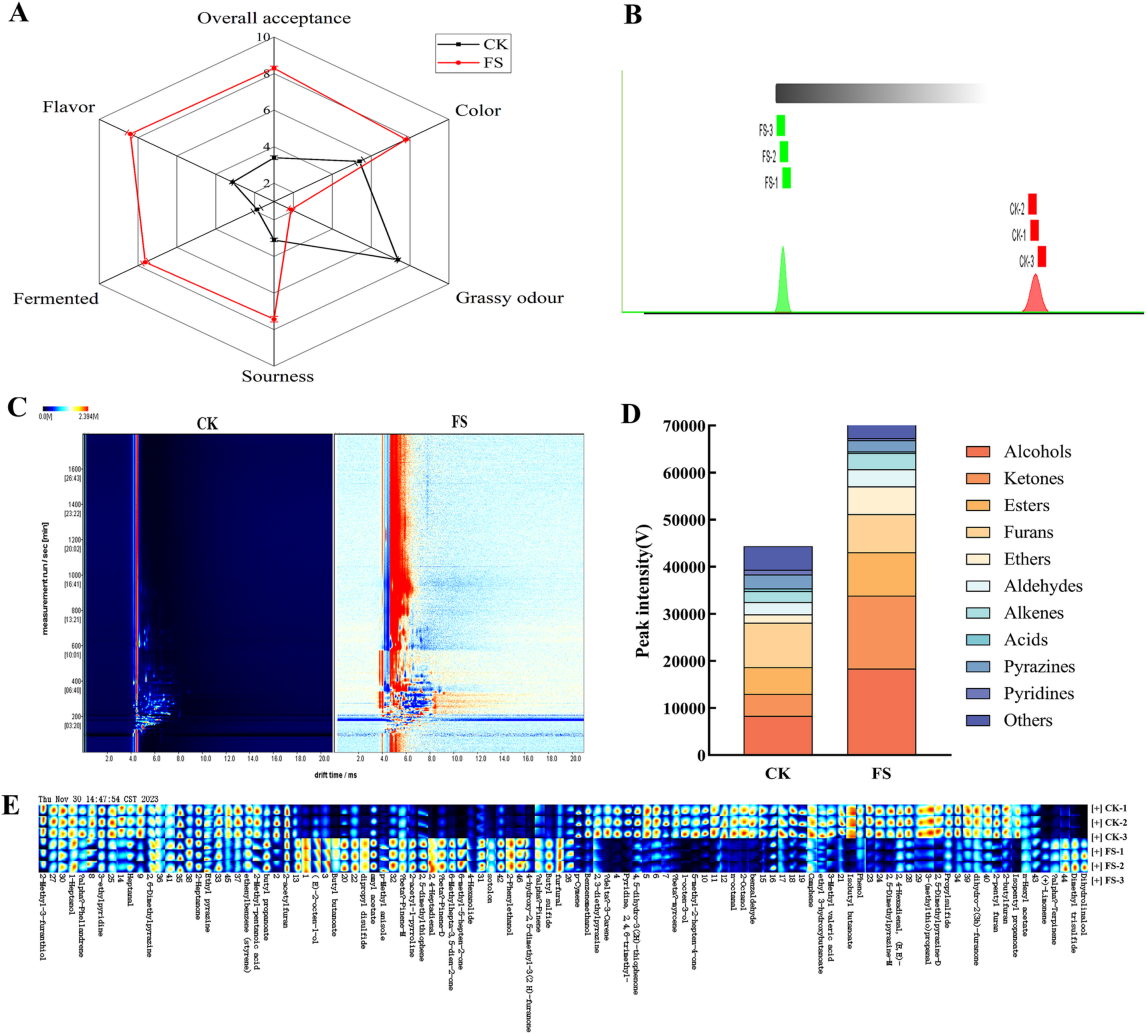
Sensory evaluation and GC-IMS observation of the supernatant before and after fermentation. **(A)** Sensory characteristics analysis radar map. **(B)** Euclidean distance diagram of nearest neighbor algorithm for different samples. **(C)** Two-dimensional difference plot. **(D)** Peak intensities of various volatile compounds. **(E)** Gallery plots indicating the variations of VOCs relative content. Panels **(C,E)** red and blue colors underline over- and under-expressed components in both.

As shown in Figure 2A, there was a significant difference in sensory rating between the CK group and the FS group (*p* < 0.05). The CK group had heavy grassy odor, which was related to the complex volatile components in mulberry leaves. Compared with CK, the color, sourness, fermented, flavor and overall acceptance of FS were significantly increased (*p* < 0.05). It may be that the sugars were converted into organic acids and aroma components during the fermentation, which contributes to the flavor formation of FS. However, the changes of volatile organic compounds before and after fermentation need further analyze to clarify the mechanism of *P. pentosaceus* JS35 fermentation on the flavor of mulberry leaves.

### 3.3 GC-IMS analysis

GC-IMS was used to analyze the differences in volatile organic compounds (VOCs) in the supernatants. Most of the signals appeared in the retention time of 200–1800 s and the drift time of 4–11 min. “Nearest neighbour” fingerprint analysis can quickly compare two groups of samples according to the intensity of the compounds in the selected evaluation area (33). The smaller the Euclidean distance between the samples, the greater the similarity, and vice versa. Figure 2B showed that the distance between the two groups was far, indicating that there was a significant difference in volatile compounds between the two groups samples. The two-dimensional difference map showed point-by-point differences between the two groups. As shown in Figure 2C, the VOCs in the two groups samples were quite different, which was consistent with the GC-IMS Euclidean distance diagram.

Fingerprint analysis showed the differences in a more intuitive way between the VOCs in the two samples. Figure 2D showed the changes of various VOCs in the supernatants before and after fermentation by *P. pentosaceus* JS35. The gallery plot showed the changes in peak intensity of the VOCs in two supernatants (Figure 2E), a total of 62 VOCs were identified in all samples (the relevant information of VOCs with VIP value >1 was shown in Supplementary Table S2), including 11 categories, namely, alcohols (8), esters (10), ketones (6), aldehydes (7), ethers (5), alkenes (10), acids (2), furans (3), pyrazines (5), pyridines (2) and others (4). In summary, the content of alcohols, esters and ketones in the supernatants were increased by *P. pentosaceus* JS35 fermentation, which effectively improved the flavor of the supernatant of mulberry leaves.

Volatile organic compounds are critical to the flavor of fermented products (34). Alcohol is an essential metabolite in the fermentation process of LAB, which not only produces flavor but also dissolves other aromatic compounds, giving fermented foods a special flavor (35). The FS had an increased content of alcohols with floral and citrus aromas, such as dihydrolinalool, 2-phenylethanol, and (E)-2-octene-1-old, which may be due to the dehydrogenation of aldehydes to alcohols during the fermentation processing. 1-octen-3-ol and 2-octano presented a grassy, pungent odor (36, 37), and their levels were reduced after fermentation from 0.74 and 0.32% to 0.08 and 0.07%, respectively, which helped to decrease the unpleasant odor of mulberry leaf.

Ester compounds are formed by the esterification of alcohols and acids. It was reported that LABs fermentation increased the content of ester compounds, which was contributing to a pronounced fruity and floral aroma (38). In this study, the relative content of esters in the supernatant increased from 6.91 to 11.26% by fermentation. Ketones contribute to fruity and sweet flavors (37). The content of ketones in the supernatant was increased sharply by fermentation, 4-hydroxy-2,5-dimethyl-3(2H)-furanone and 6-methylhepta-3,5-dien-2-one are ketones with caramel and coconut sweet aromas, which increased by 7.11-fold and 7.55-fold, respectively. These above compounds enriched in the fermented supernatant and contributed the sweet aroma.

The relative contents of ethers, aldehydes and alkenes in the fermented supernatant were low, but the thresholds of ethers and aldehydes were very low, which make them to be important flavor compounds (37). Compared with CK, the total amount of ether compounds of FS increased by 2.32-fold, especially the contents of *p*-methyl anisole and butyl sulfide with rose fragrance increased by 2.32- and 3.21-fold, respectively. 2,4-Heptadienal is an aldehyde with a fruity flavor, and its content increased by 9.40-fold after fermentation. (+)-Limonene, an alkene compound with a fresh aroma, increased in relative content from 0.26 to 1.47% after fermentation. It has reported that LAB fermentation effectively improved the sensory quality of mulberry leaves by reducing the grassy flavor and increasing floral flavor (36). Our results also showed that FS had pleasant characteristic flavors having floral, fruity and minty aromas, while reduced the grassy and earthy flavors of mulberry leaves. In summary, *P. pentosaceus* JS35 has excellent potential to improve the flavor of mulberry leaf products.

### 3.4 Non-targeted metabolomics to analysis differential metabolites and metabolic pathways

#### 3.4.1 Differential metabolite screening and analysis

**Figure 3:**
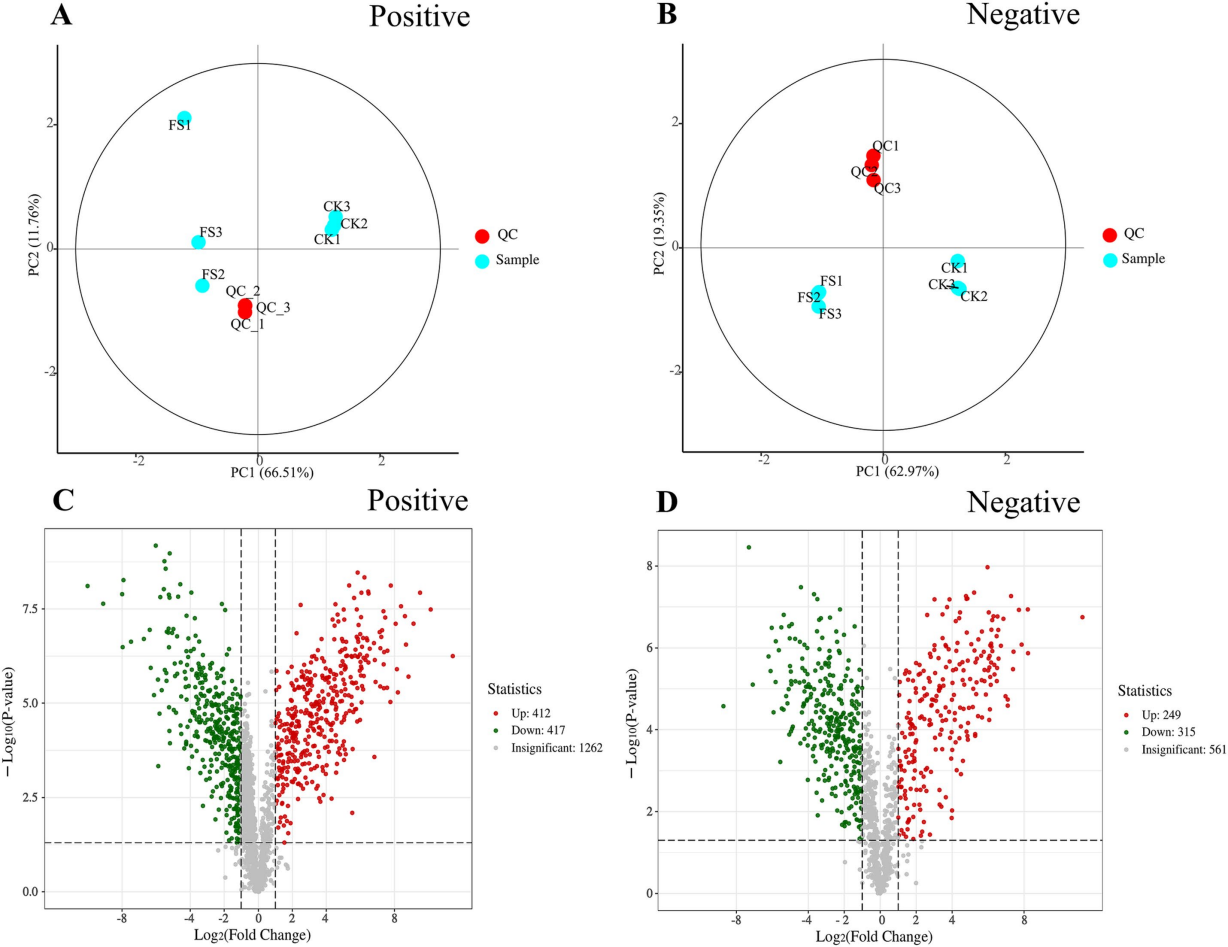
Effects of non-volatile compounds in fermentation supernatant before and after fermentation. **(A)** PCA score plot of CK, FS and QC samples in positive ion mode. **(B)** PCA score plot of CK, FS and QC samples in negative ion mode. **(C)** Volcano diagram of metabolites in positive ion mode. **(D)** Volcano diagram of metabolites in negative ion mode.

Non-targeted metabolomics was used to detect metabolites in different samples. Principal component analysis (PCA) was used to evaluate the metabolite profile. As shown in Figures 3A,B, the data points for the QC group are closely distributed. The three biological replicates for the CK and FS samples were tightly clustered in the score plot, indicating that the results were reproducible and reliable (13). As shown in Figures 3C,D, a total of 3,216 metabolites in supernatants were identified. The gray dots indicated metabolites that are not significantly different in the CK supernatant and the FS, and the red dots and green dots indicate the metabolites that are significantly up-regulated and down-regulated, respectively. In the positive ion mode, CK and FS identified a total of 2091 metabolites (412 up-regulated, 417 down-regulated). In the negative ion mode, CK and FS identified a total of 1,125 metabolites (249 up-regulated, 315 down-regulated).

**Figure 4:**
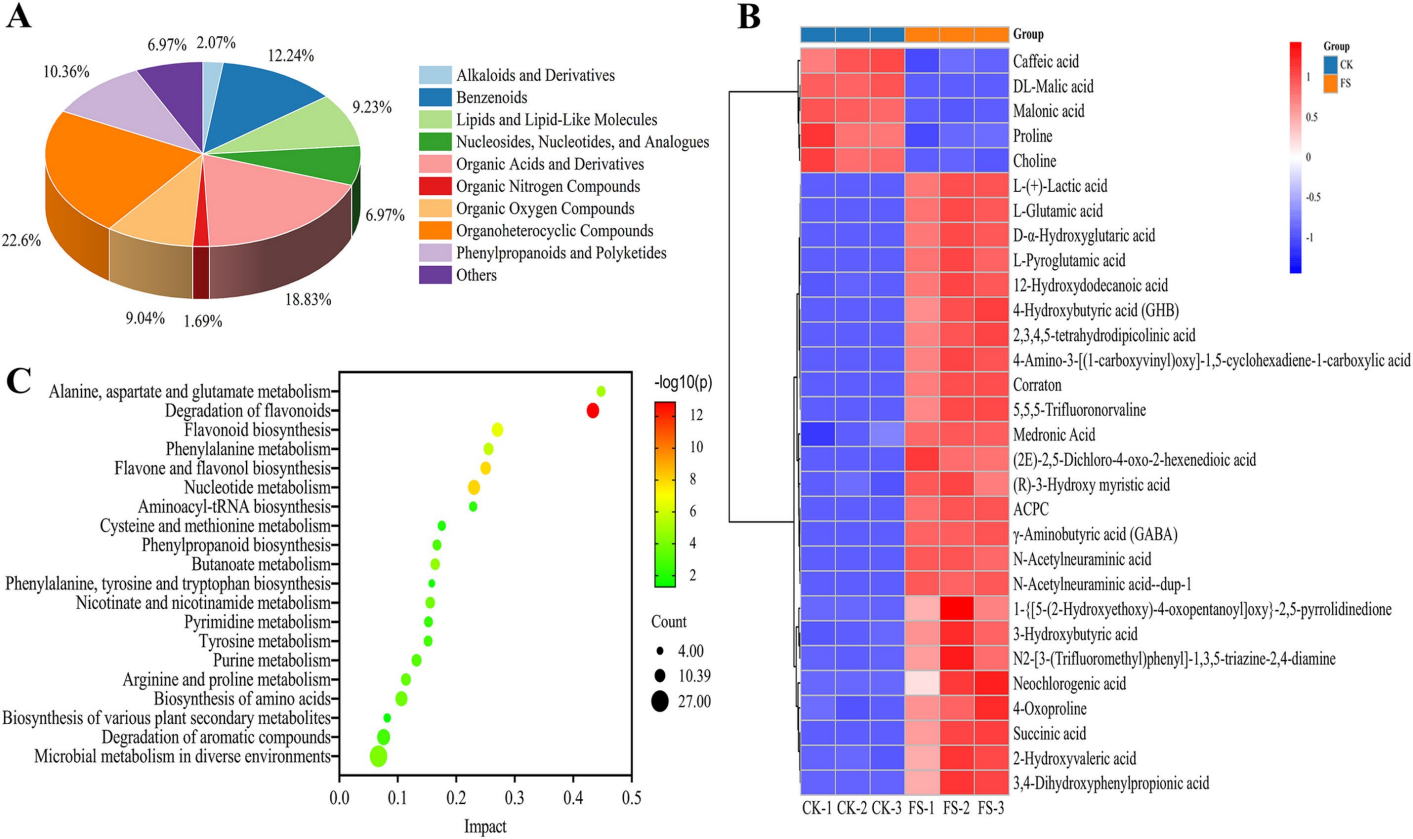
Analysis of differentially expressed metabolites in fermentation supernatant before and after fermentation. **(A)** Classification of differential metabolites. **(B)** Cluster analysis of the top 30 differential metabolites. **(C)** Bubble diagram of the metabolic pathway enrichment.

The variable influence on projection (VIP) score, *p*-value, and fold change (FC) were used to screen the difference metabolites. The following criteria were met for screening: |log2 FC| > 1, VIP > 1 and *p* < 0.05. A total of 531 differential metabolites were screened (283 up-regulated, 248 down-regulated). The metabolites were categorized into 13 different classes, including organoheterocyclic compounds (22.60%), organic acids and derivatives (18.83%), benzenoids (12.24%), phenylpropanoids and polyketides (10.36%) and lipids and lipid-like molecules (9.23%) and others (Figure 4A). It was reported that microbial fermentation can alter the metabolic profile of food material. Meng et al. (39) found that different LAB cultures fermented loquat juice presented different metabolic profile. Organoheterocyclic compounds, organic acids and derivatives were abundant in FS, which play key roles in the flavor of fermentation supernatant (40). In this study, the top 30 differential metabolites in abundance were selected for cluster analysis (Figure 4B and Supplementary Table S3). In Figure 4B, CK and FS were clearly presented into two different metabolic profiles, and further highlighted that LAB fermentation significantly altered the metabolic characteristics of mulberry leaf. L-(+)-lactic acid content was up-regulated 6.97-fold while malic acid content was down-regulated by 89.71%. The changes of biosynthetic pathway of sugars and LAB metabolism may be lead the malic-lactic acid conversion during fermentation (41). L-glutamic acid and GABA contents were up-regulated by 7.71- and 6.00-fold, respectively. *P. pentosaceus* JS35 strain is a GABA-producing strain, and use glutamate or its sodium salt to convert into GABA, resulting in an up-regulation of GABA. Neochlorogenic acid, a phenolic compound, having excellent antioxidant capacity, was up-regulated 1.59-fold after fermentation. Conversely, with the progress of the fermentation, some flavonoids or phenolic acids were degraded, such as caffeic acid. The possible reason is that the compounds were used as carbon sources by LABs fermentation (32).

#### 3.4.2 KEGG enrichment analysis

Through pathway enrichment analysis, 124 differential metabolic pathways were obtained. The top 20 metabolic pathways were shown in Figure 4C. The top 5 metabolic pathways were as follows: (1) Degradation of flavonoids; 14 metabolites such as daidzein, quercetin-3β-d-glucoside, kaempferol and naringenin were found in this pathway. (2) Nucleotide metabolism; 13 metabolites such as 2′-deoxyadenosine, adenosine and guanine were found in this pathway. (3) Flavone and flavonol biosynthesis; 10 metabolites such as apigenin, quercitrin and quercetin 3-*O*-rhamnoside-7-O-glucoside were found in this pathway. (4) Flavonoid biosynthesis; 12 metabolites such as kaempferol, quercetin, taxifolin and neohesperidin were found in this pathway. (5) Phenylalanine metabolism, 9 metabolites such as l-phenylalanine, *trans*-cinnamic acid and succinic acid were found in this pathway. Among the above pathways, metabolites including daidzein, quercetin-3β-d-glucoside, quercetin, kaempferol, naringenin, and trans-cinnamic acid were significantly increased which were proved having antioxidant capacity (42, 43). Essential amino acids such as phenylalanine, tyrosine, and tryptophan were significantly increased. That is to say, the fermentation by *P. pentosaceus* JS35 also enhanced the nutritional value and antioxidant capacity of mulberry leaf.

### 3.5 Antioxidant capacity of supernatants *in vitro*

**Figure 5:**
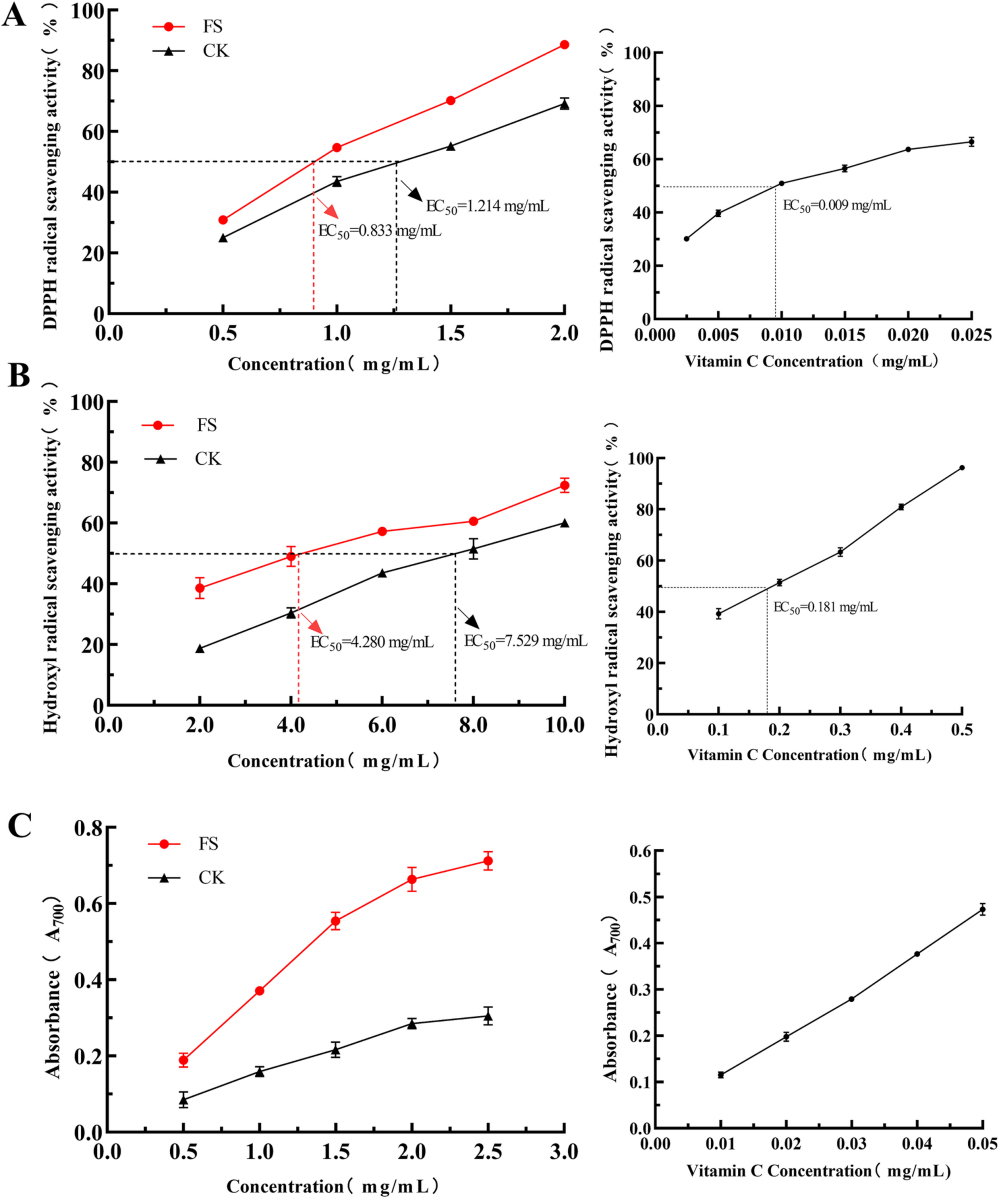
Effect of FS on antioxidant capacity. **(A)** The DPPH radical scavenging capacity. **(B)** Hydroxyl radical scavenging capacity. **(C)** The reducing ability. Results are expressed as the mean ± standard deviation (*n* = 3).

The DPPH free radical scavenging ability is the most widely used indicator of the antioxidant properties of food materials. As shown in Figure 5A, with increasing of supernatant concentration, the DPPH free radical scavenging activity gradually increased, and the two were positively correlated. The EC_50_ value of the FS for DPPH free radical were 0.833 mg/mL, while that of the CK was 1.214 mg/mL, respectively. At a concentration of 2 mg/mL, the DPPH free radical scavenging ability of CK and FS was 69.21 ± 1.80% and 88.62 ± 1.06%, respectively. Abbas et al. (44) used *Bacillus subtilis* H4 (H4) and *Bacillus amyloliquefaciens* LFB112 bacterial strains for submerged fermentation to enhance the antioxidant capacity of mulberry-derived postbiotics (MDP). The results showed that the antioxidant capacity of MDP was significantly improved after fermentation. When the concentration was 2 mg/mL, the DPPH free radical scavenging ability of unfermented mulberry leaves and MDP was 70.68 and 90.95%, respectively (44).

In various reactive oxygen species, hydroxyl radicals are the most reactive and have the capacity to react with a wide range of biomolecules within living cells, ultimately leading to cell death and tissue damage (45). As shown in Figure 5B, FS had better scavenging abilities against hydroxyl radicals. The EC_50_ value of FS was 4.280 mg/mL, while that of CK was 7.529 mg/mL.

The total reducing power is another commonly used indicator to evaluate the activity of antioxidants. Compared with the CK, the reducing capacity of FS was significantly increased (*p* < 0.05). As the supernatant concentration increased, the reducing capacity gradually increased, showing a dose-dependent relationship (Figure 5C). When the concentration was 2.5 mg/mL, the total reducing power of CK and FS was 0.31 ± 0.02 and 0.71 ± 0.02, respectively. Chen et al. (13) used mixed strains to solid-state fermentation of mulberry leaves. The results showed that when the concentration was 4 mg/mL, the reducing power values of the extracts of unfermented and fermented mulberry leaves were 0.440 and 0.589, respectively (13). Polyphenols are a class of active substances with excellent antioxidant capacity, and their content is usually closely related to its antioxidant capacity (46). The total phenolic content increased by 35.29% in FS (Figure 1C). Non-targeted metabolomics analysis also showed that more phenolic compounds related to antioxidant capacity were synthesized, such as neochlorogenic acid, quercetin and kaempferol. In summary, the contents of the active compounds were consistent with the antioxidant capacity.

### 3.6 Antioxidant capacity of FS in *C. elegans*

**Figure 6:**
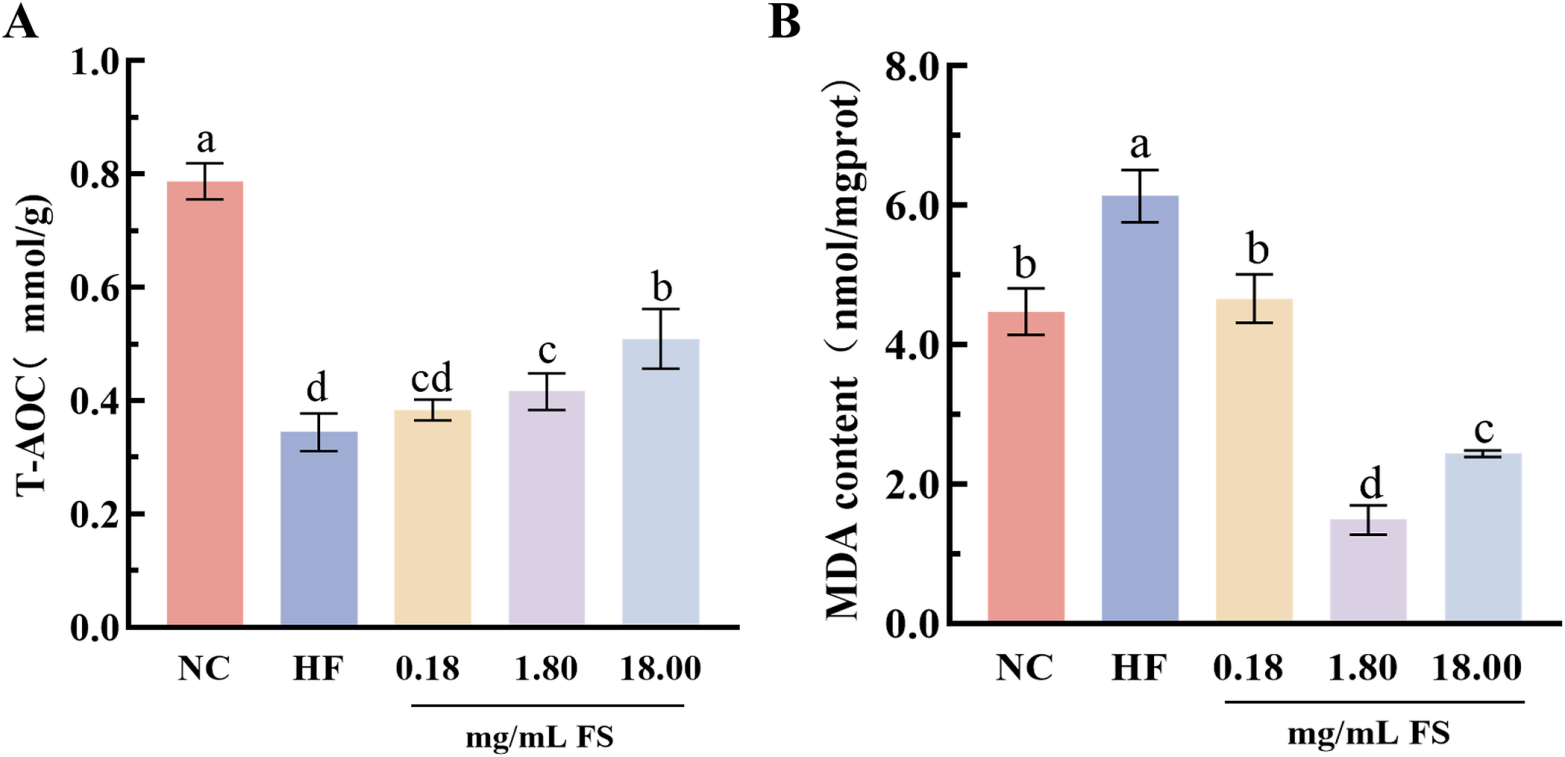
Effects of FS on biochemical indicators of *C. elegans*. **(A)** Total antioxidant capacity. **(B)** Malondialdehyde content. Results are expressed as the mean ± standard deviation. Means with different letters in figure were significantly different at *p* < 0.05.

*Caenorhabditis elegans* is a commonly utilized model organism for assessing functional attributes, including anti-aging and antioxidant capacities (47). In this study, the high-fat *C. elegans* was employed to assess the *in vivo* antioxidant properties of FS. T-AOC is the sum of various antioxidant capacities, which can reflect the capacity of the antioxidant system (48). As shown in Figure 6A, high-fat diet treatment reduced the activity of total antioxidant capacity in *C. elegans*, and its activity was significantly increased after the addition of FS in diet. Compared with the HF group, the total antioxidant capacities of 0.18, 1.8, and 18 mg/mL FS treated *C. elegans* significantly increased by 11.76, 23.53, and 50.00%, respectively.

The MDA is one of the free products of polyunsaturated fatty acid peroxidation in cells, which was often used to evaluate the degree of lipid peroxidation and oxidative stress in the body (49). The higher the content of MDA, the more severe the oxidative damage in the body. The MDA content of *C. elegans* in the HF group was substantially higher than that of the NC group (Figure 6B), Indicating that a high-fat diet led to a decrease in the ability of *C. elegans* to resist lipid peroxidation. The MDA content of *C. elegans* treated with FS was significantly reduced (*p* < 0.05), especially, that of the 1.80 mg/mL FS treatment was significantly reduced by 75.69% (Figure 6B). This may be because the FS are rich in GABA, flavonoids, polyphenolics and other active components, which have anti-lipid peroxidation capacities. Xiao et al. (50) reported that GABA-rich fermentation supernatant improved cellular oxidative stress by reducing ROS and MDA content. It was demonstrated that dietary FS significantly increased the antioxidant capacity of *C. elegans* and protected cells from oxidative damage, but the further mechanism still remains to be investigated.

### 3.7 Effects of FS on basic physiological indices of *C. elegans*

**Figure 7:**
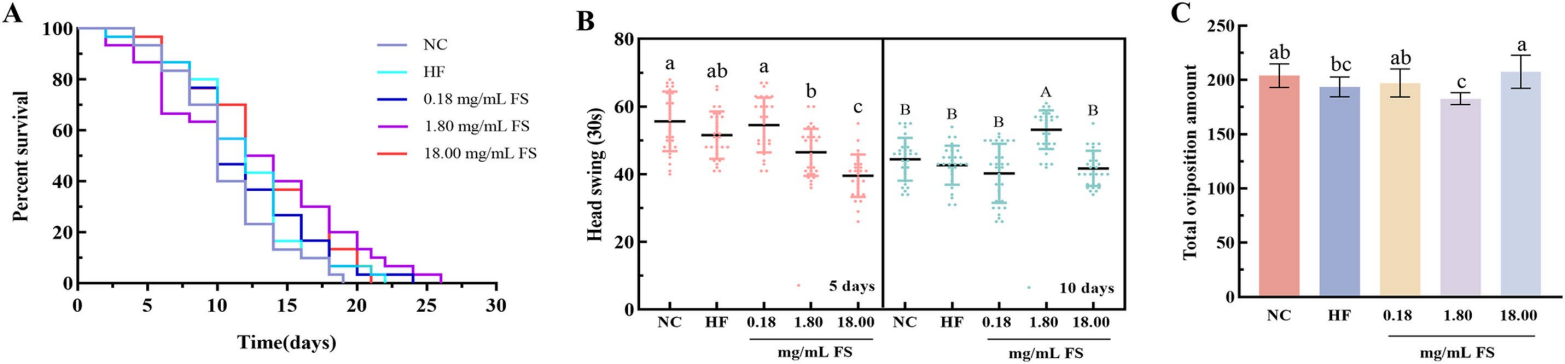
Effects of FS on basic physiological indicators of *C. elegans*. **(A)** Survival curves. **(B)** Head swing. **(C)** Total oviposition amount. Results are expressed as the mean ± standard deviation (**A**, *n* = 90; **B**, *n* = 30; **C**, *n* = 10). Means with different letters in figure were significantly different at *p* < 0.05.

In order to explore the effects of FS on the life and health of *C. elegans*, the related physiological indices of *C. elegans* were determined. The extended lifespan of *C. elegans* is accompanied by an improvement in overall health (51). As shown in Figure 7A and Table 1, compared to the HF group, the average lifespan of *C. elegans* in the FS treatment groups (0.18, 1.80, and 18.00 mg/mL) was significantly prolonged, increasing by 7.17, 17.37, and 10.46%, respectively (*p* < 0.05). Among them, the maximum lifespan of the 1.80 mg/mL FS treatment group reached 23.67 ± 0.58 days. The effects of FS on prolonging life may be related to the active components of mulberry leaves or metabolites produced by *P. pentosaceus* JS35 fermentation. It was reported that some active ingredients, such as phenolics, polysaccharide and GABA, alleviated exogenous stress conditions and prolonged the lifespan of *C. elegans* (52). In this study, FS had a dose-dependent effect on prolonging the lifespan of *C. elegans*, and 1.80 mg/mL FS prolonged the lifespan mostly in all groups.

**Table 1:** The effect on mean lifespan and maximum lifespan of *C. elegans* after FS treatment.

Lifespan (d)	NC	HF	0.18 mg/mL	1.80 mg/mL	18.00 mg/mL
Mean	10.78 ± 0.48^d^	11.57 ± 0.62^cd^	12.40 ± 0.49^bc^	13.58 ± 0.10^a^	12.78 ± 0.25^ab^
Maximum	19.00 ± 1.00^C^	21.00 ± 1.00^B^	21.67 ± 0.58^B^	23.67 ± 0.58^A^	20.00 ± 1.00^BC^

Data expressed as mean ± Standard deviation (*n* = 3). Different superscript letters in the same column indicate differences (*p* < 0.05).

Mobility represents energy consumption. The higher frequency of exercise, the more energy consumption, and the lower degree of fat accumulation (53). The head swing frequency of *C. elegans* was determined in this study, and the results were showed in Figure 7B. On the 5th day, 0.18 mg/mL FS treatment significantly increased the head swing frequency of the *C. elegans*. On the 10th day, the 1.80 mg/mL FS treatment significantly increased the head swing frequency of the *C. elegans* (*p* < 0.05).

The oviposition ability of *C. elegans* is related to its growth and development. Compared with the HF group, the oviposition of *C. elegans* treated with 18.00 mg/mL FS increased by 7.23%, and that of the *C. elegans* treated with 1.80 mg/mL FS decreased without significant difference (*p* > 0.05, Figure 7C). It has been reported that some active substances extended life but at the same time reduced the body’s reproductive capacity, which was a balance mechanism (54). In this study, the supplement of FS extended the lifespan of *C. elegans* without causing damage to the reproductive capacity. Similar results were also reported that the longevity of *C. elegans* was prolonged by some phenolics without affecting egg production (26).

## 4 Conclusion

The fermentation by *P. pentosaceus* JS35 significantly improved the flavor, metabolic profile, and antioxidant activity of mulberry leaves. After *P. pentosaceus* JS35 fermentation, the contents of GABA and total phenols in the supernatants were increased significantly, the contents of favorite flavor compounds such as dihydrolinalool, 2-phenylethanol and *p*-methyl anisole were increased significantly and the contents of unpleasant flavor compounds such as 1-octen-3-ol and 2-octano were significantly decreased. The metabolic profile of mulberry leaves extracts mainly flavonoids metabolism significantly changed, especially kaempferol, quercetin, myricetin, and daidzein in FS significantly increased. The FS has good antioxidant capacity both *in vitro* and in *C. elegans*. The EC_50_ values of the fermentation supernatant for DPPH free radical and hydroxyl radical scavenging abilities were 0.833 and 4.280 mg/mL, respectively. The FS was dietary supplemented to high-diet *C. elegans*, and effectively increased its T-AOC level and decreased MDA level, finally prolonged the lifespan. This study provides a thread for the further application of the *P. pentosaceus* JS35 strain in the processing of fermented products, and the fermented supernatant of mulberry leaf is potential as a functional drinks or food ingredients.

## Data Availability

The original contributions presented in the study are included in the article/Supplementary material, further inquiries can be directed to the corresponding author.
