# Supplementary material for: Pediococcus pentosaceus JS35 improved flavor, metabolic profile of fermentation supernatant of mulberry leaf powder and increased its antioxidant capacity
Source: Front Nutr. 2025 Mar 4;12:1551689. doi: 10.3389/fnut.2025.1551689 (PMC11913688; doi:10.3389/fnut.2025.1551689)
Supplement: Supplementary file 1 [file Table_1.docx]

**Supplementary Table 1**

Sensory evaluation of supernatant before and after fermentation.

| Category | Description | Score |
| --- | --- | --- |
| Color | The color is yellowish brown, clear and transparent, shiny. | 0-10 |
| Flavor | The aroma is strong, without any odor. | 0-10 |
| Sourness | Moderate acidity. | 0-10 |
| Fermented | The fermentation taste and flavor are moderate. | 0-10 |
| Grassy odor | The grassy odor is strong and has a pungent odor. | 0-10 |
| Overall acceptance | Completely acceptable. | 0-10 |

Note: 0-3: The characteristic was hard to observe; 4-6: The characteristic could be observed mediumly; 7-10: The characteristic could be observed strongly.
